# Mental health symptoms among homeless shelter residents during COVID-19 lockdown in Tshwane, South Africa

**DOI:** 10.4102/phcfm.v15i1.3730

**Published:** 2023-04-03

**Authors:** Joanelle Stonehouse, Gerhard Grobler, Urvisha Bhoora, Michelle N.S. Janse van Rensburg

**Affiliations:** 1 Department of Family Medicine, Faculty of Health Sciences, School of Medicine, University of Pretoria, Tshwane, South Africa; 2 Department of Psychiatry, Steve Biko Academic Hospital, Faculty of Health Sciences, University of Pretoria, Tshwane, South Africa

**Keywords:** mental health, homelessness, temporary shelters, COVID-19 lockdown, substance use, opioid withdrawal

## Abstract

**Background:**

In order to contain the spread of COVID-19 in South Africa during the national state of emergency, the Gauteng Department of Social Development established temporary shelters and activated existing facilities to provide basic needs to street-homeless people in Tshwane, which facilitated primary health care service-delivery to this community.

**Aim:**

This study aimed to determine and analyse the prevalence of mental health symptoms and demographic characteristics among street-homeless people living in Tshwane’s shelters during lockdown.

**Setting:**

Homeless shelters set up in Tshwane during level 5 of the COVID-19 lockdown in South Africa.

**Methods:**

A cross-sectional, analytical study was conducted using a *Diagnostic and Statistical Manual of Mental Disorders* (DSM-5)-based questionnaire that looked at 13 mental health symptom domains.

**Results:**

Presence of moderate-to-severe symptoms were reported among the 295 participants as follows: substance use 202 (68%), anxiety 156 (53%), personality functioning 132 (44%), depression 85 (29%), sleep problems 77 (26%), somatic symptoms 69 (23%), anger 62 (21%), repetitive thoughts and behaviours 60 (20%), dissociation 55 (19%), mania 54 (18%), suicidal ideation 36 (12%), memory 33 (11%) and psychosis 23 (8%).

**Conclusion:**

A high burden of mental health symptoms was identified. Community-oriented and person-centred health services with clear care-coordination pathways are required to understand and overcome the barriers street-homeless people face in accessing health and social services.

**Contribution:**

This study determined the prevalence of mental health symptoms within the street-based population in Tshwane, which has not previously been studied.

## Introduction

The street-homeless population worldwide is at an increased risk of mental illness and presentation to healthcare facilities in this vulnerable community is often acute, of more intense severity, and more complex because of limited access to mental healthcare services.^1^ This research aimed to determine and describe the prevalence of mental health symptoms and analyse selected demographic characteristics among street-homeless people living in shelters during the national coronavirus disease 2019 (COVID-19) lockdown in an urban setting in the city of Tshwane (CoT), South Africa. This provided the opportunity to identify and promote improvements in quality of healthcare and specifically mental health services, rendered to this population.

### Homelessness: A global reality

The United Nations projects that by 2050 the world’s population will have grown to more than 9.6 billion people, with the largest growth expected to be in Africa, and a large number of these people will be living in urban areas (an estimated 70%).^2^ Urbanisation itself gives rise to other problems, with one of the most important social and economic challenges being displacement. By the end of the 20th century, homelessness was accepted to be a complicated phenomenon and an extreme form of social exclusion, rather than previous notions that homelessness is a lifestyle choice. Thus, according to Tipple and Speak, also quoted by Kriel, a single definition of homelessness ‘may be inappropriate and … a range of definitions may be needed to underpin interventions and policy development’.^2^

It is important to distinguish between the terms ‘homeless’ and ‘homelessness’. Atlanta Mission, a North American association united in ending homelessness, suggests that homelessness is what an individual’s circumstances led them to. It is what they are going through, rather than who they are:

When we use the term ‘homeless’, we’re implying that there is no hope for change. But when we say someone is currently ‘experiencing homelessness’, we’re implying that it’s something they won’t experience forever.^3 (p.1)^

### Homelessness in South Africa

The number of street-based homeless people in South Africa is estimated to be between 100 000 and 200 000 people. Numbers are unpredictable and based on assumptions, despite some research, as it is difficult to accurately quantify numbers because of the high mobility of this community. Also, the composition of the homeless population in South Africa varies and the stereotype of only men being street dwellers has long been proven wrong. Even though homelessness affects all races in South Africa, black Africans make up the largest percentage. South African citizens and non-citizens (who may have left their countries because of political or socio-economic reasons) are found among this population, with women and children being the most vulnerable within this group.^4^

Homelessness in South Africa has multiple core origins, including historical policies as well as social and economic contributors. Poverty, secondary to unemployment, is a major contributor, also as a result of rural-urban migration for job seeking opportunities. Social contributors include interpersonal violence in the home, divorce and single parents or child-headed homes because of health-related problems such as human immunodeficiency virus and acquired immunodeficiency syndrome (HIV/AIDS).^4,5^ Substance use and substance use disorders often exacerbate the above-mentioned contributors to homelessness and can lead to homelessness itself.^6^

### Substance use in the local context

The National Coalition for the Homeless agree that some people are homeless because of substance use, and some use substances to cope with their circumstances and experiences.^4^ Together with tobacco and cannabis, the most common substance being used is a heroin-containing ‘cocktail’ mixture, known on the streets as ‘nyaope’ or ‘whoonga’. A study conducted in four large hospitals in the CoT in 2017 showed that 11 of 401 respondents (3%) reported having ever used a non-regulated opioid (specifically heroin). Eight respondents had used a non-regulated opioid in the previous six months, of which seven had injected the opioid.^7^ Besides the psychomimetic effects of the drugs, members of the street-homeless community may also be at higher risk of mental health conditions.^1^

### Mental health conditions and the street-homeless community

Street-homeless communities experience a high burden of mental illness. Hospitalisation for psychiatric conditions in the street-homeless community are often of longer duration and have a higher financial burden than for the general population as a result of more severe and complex psychiatric illness.^1^

Attendance of appointments at community-based clinics for the outpatient treatment of mental health conditions plays a cardinal role in improved mental health for mental healthcare users (MHCUs). Appointments that are missed or not adhered to may have detrimental consequences to the treatment programme of MHCUs. So much more so, access to screening and diagnosis of mental health conditions, even before acute admission, is indicated because of lacking outpatient community-based services.^8^

Another barrier contributing to whether care is sought or not is how a person perceives their need for care. Not all people who meet the medical criteria for mental health treatment or intervention realise that they experience sufficient impairment or disability or meet the criteria for intervention. Experiencing unmet needs for health or social services seems to increase with socio-economic marginalisation. Street-homeless people who use drugs experience a high rate of perceived unmet needs because of inaccessible mental health and substance use healthcare services.^9^

### Coronavirus disease 2019 national lockdown

As part of the national response to contain the spread of COVID-19 during level 5 lockdown from 26 March 2020 in South Africa, the Department of Social Development established new shelters and activated existing shelters to provide accommodation and food to the street-homeless community in the CoT.^10^ The placement of street-homeless people into shelters gave rise to the opportunity for provision of healthcare services to this vulnerable community.

The University of Pretoria’s (UP) Department of Family Medicine took initiative to respond to the need for provision of essential primary health care services during this difficult time. Seven priorities for action were identified by the team, related to COVID-19 and non-related conditions. The COVID-19 symptom screening at health facilities for non-acute patients: (1) ensure accurate and effective information flows between staff at all levels of service; (2) develop new COVID-19 standard operating procedures (SOPs); (3) train staff to implement COVID-19 SOP’s; (4) sustain essential primary care services for non- COVID-19 conditions; (5) support and provide services to homeless people and those living in shelters; and (6) develop COVID-19 literacy.^11^ Despite many challenges faced, the needs of homeless people in the CoT were addressed as best as possible by collaborative teams during a state of emergency. Coronavirus disease 2019 screening and primary health care services were provided at the shelters in conjunction with various stakeholders, such as the Community Oriented Substance Use Programme (COSUP) and several non-governmental organisations (NGOs) that have been involved in the support of the street-homeless community in and around Tshwane, even before the pandemic.^11^

Community Oriented Substance Use Programme^12^ uses the Community Oriented Primary Care (COPC) approach to health and well-being, mainly to provide services to people who use drugs. Community Oriented Primary Care mobilises health resources in the community where people work and live and brings public health and clinical care together to people in their communities. Community Oriented Primary Care is based on five principles, namely local health and institutional analysis, comprehensive care, equity of care, practicing with science and service integration around users.^13,14^

Opioid substitution therapy (OST) and needle and syringe programmes (NSPs) are main harm reduction services provided at COSUP sites. Other services provided at the sites include psycho-social services and skills development, thus creating a positively enabling environment to a vulnerable community.^12,14^

Service-users visiting COSUP and other service-providers, who seek multidisciplinary services, including NSP, primary health care and social support services, are at the point of care. Frequent interaction with staff builds trusted relationships and is an opportunity for the possible identification of underlying mental healthcare concerns and appropriate referral. Harm reduction practices at the point of contact and healthcare are of crucial importance, especially for those seeking healthcare who are substance dependent. Services rendered by interdisciplinary teams will be beneficial and facilitate opportunities for a better chance in life. Benefits include improved general and mental health with decreased hospitalisation and costs to the state.^9,13,14,15^

The Tshwane Leadership Foundation (TLF) is an organisation committed to transformation in the inner-city community, and they have been assisting the homeless and destitute population of Tshwane since 1993. Tshwane Leadership Foundation runs projects addressing problems related to poverty, such as social housing, palliative care services, skills training, advocacy and outreach work. For those requiring chronic medication and follow-up, transport services are made available for hospital or clinic visits at nearby facilities.^16^ Pretoria Evangelism and Nurture (PEN), working in the inner-city since 1992, in partnership with Tshwane Homeless Forum set up 11 temporary shelters during the lockdown period, making space for almost 350 street-homeless people, ensuring safe accommodation with regard to requirements and protocols for COVID-19.

The setting up of shelters for the street-homeless community during the pandemic lockdown gave rise to the opportunity for the identification of major burdens of disease and contributing factors. Even though studies on the prevalence of mental health conditions among the street-based community are limited, this study’s findings suggest that there is a major need for specialised mental healthcare interventions for members of the homeless community.^17^

## Research method and design

### Study design

A cross-sectional, analytical study was conducted to determine the prevalence of mental health symptoms among the street-homeless community accommodated in temporary homeless shelters across Tshwane during the national state of emergency and COVID-19 level 5 lockdown.

### Setting

Several temporary homeless shelters were set up by the CoT, and various NGOs activated existing shelters during level 5 of the COVID-19 lockdown (see Table 1). Around 25 shelters were active throughout the lockdown period in and around the city centre of Tshwane, of which eight shelters were managed by the CoT, and the others were managed by churches and NGOs. Shelter data and reports of nine shelters were shared with care providers and stakeholders alike by the CoT. Facility details and number of people accommodated for the shelters that made data available is shared in Table 1. Medical services were provided at these shelters, and therefore these nine shelters were accessed for data collection.

**TABLE 1:** Temporary homeless shelter information.

Shelter	Institution responsible	Gender of residents accommodated	Number of people accommodated†
Lyttelton Sportpark Shelter (Temporary tented shelter)	CoT	Males	348
Lyttelton Town Hall Shelter (Temporary shelter in town hall facility)	NGO	Males	31
Lyttelton Art Gallery Shelter (Temporary shelter in art gallery facility)	CoT	Males and females	21
Potter’s House (Transitional housing)	NGO	Females	41
Capital Park Shelter (Temporary tented shelter)	CoT	Males	52
St Wilfrid’s Anglican Church Shelter (Temporary shelter in church hall)	Church	Males	24
Danville Shelter (Lucas van Der Berg Stadium) (Temporary shelter)	CoT	Males and females	400
Akasia Town Hall Shelter (Temporary shelter within town hall facility)	CoT	Males	33
Akasia Life Changing Ministries Church (Temporary tented shelter)	Church	Males	90

CoT, City of Tshwane; NGO, non-governmental organisations.

† , Data obtained from Marcus TS, Heese J, Scheibe A, Shelly S, Lalla SX, Hugo JF. Harm reduction in an emergency response to homelessness during South Africa’s COVID-19 lockdown. Harm Reduct J. 2020;17(1):60. https://doi.org/10.1186/s12954-020-00404-0

### Study population

Our study population was street-homeless people living in temporary homeless shelters set up by the CoT, churches and NGOs during the COVID-19 level 5 lockdown. Medical services were provided in the shelters, which included primary health care, screening for COVID-19 and mental health symptoms, as well as opioid substitution therapy (withdrawal management). This carried the advantage of a study population, not previously accessible, conveniently being gathered together in the shelter settings because of strict lockdown regulations.

### Intended sample size

The intended sample size was calculated with the assistance of a biostatistician. Intended sample size for this study depended on the number of shelter inhabitants who volunteered to participate during data collection. Table 2 displays the required sample sizes associated with a particular expected prevalence and the accuracy to which the latter needs to be estimated. A high frequency of prevalence of mental health conditions for individuals was expected, for example, 75%, and a sample of at least 289 participants was required to estimate the expected prevalence to an accuracy within 5%. The aim was to enrol about 300 participants.

**TABLE 2:** Intended sample size.

Prevalence (%)	Accuracy		
	10%	7.5%	5%
65	88	156	350
70	81	144	323
75	73	129	289
80	63	110	246

### Sampling method

As a result of the chaotic manner in which shelters were set up in the early stages of the emergency response and the rapid turnover of residents accommodated in temporary shelters, convenience sampling was used to collect data. This vulnerable population would likely not otherwise be available and accessible for data collection.

Street-homeless residents in temporary homeless shelters who were willing to participate and capable of giving informed consent were asked to participate. Interviews were conducted at different times on different days and at different shelters to include as many participants as possible to reach an appropriate sample size as calculated by the biostatistician. All participants’ available data were included in the sample for analysis.

### Data collection

Residents in the temporary homeless shelters were asked to complete a questionnaire during an interview as part of medical assessment. Written informed consent was obtained from each individual participant. Individuals who were unable to give consent were not included. To reduce systematic errors, interviews were conducted by the first author (a medical doctor and family medicine registrar) and four final-year clinical associate (BCMP) students completing their mental health rotations. The first author and research assistants had been trained in using the DSM-5 Cross-Cutting (CC) Symptom Measure via Zoom by the third author, a psychiatrist (Head of the Psychiatry clinical unit at an academic institution) who is familiar with the tool.

The DSM-5 CC Symptoms Measure was developed by the DSM-5 Task Force and Work Groups to serve as an assessment for the presence and severity of 13 psychiatric symptom domains measured over 2 weeks. Domains assessed include depression, anger, mania, anxiety, somatic symptoms, suicidal thoughts, psychosis, sleep disturbance, cognition and/or memory problems, obsessive thoughts and behaviours, dissociation, personality functioning and substance use in adults. The tool consists of 23 questions for adults, using a five-point scale to indicate severity of symptoms.^18^

The advantages of the DSM-5 CC measure are that it is easy to complete, score and interpret. The assessment is freely available for download and use with permission from the American Psychiatric Association. Patients are able to self-report the measures, therefore engaging in their own assessment and care. Even though dimensional measurements in psychiatry are not a standard practice, the use and availability of these measures provide a standardised way for clinicians to assess patients over time. Clinicians’ education on the proper use and interpretation of the tool is essential, as it is not intended to be used as screening for specific disorders only. The focus should rather be to use it at the initial patient interview and to redo the symptom measure at follow-up appointments to monitor treatment progress, regardless of a possible specific DSM diagnosis.^18^

### Data analysis

Questionnaires were reviewed for completeness and unanswered questions were asked to the participant again and completed. Data from the questionnaires were entered into an Excel spreadsheet with a numbering system to maintain confidentiality. If not complete, recipient data were not entered into the Excel spreadsheet. The data from the Excel database were used to conduct this study.

Data were analysed by a biostatistician. Analysis of the overall prevalence of mental health symptoms was carried out for individuals. This was reported as a percentage along with a 95% confidence interval (CI). Estimation of the latter was performed with svy prefix in Stata. The frequency and 95% CI was determined for each of the 13 domains, which was relevant to describe the most prevalent mental health symptom domains among street-homeless people. For each of the 13 domains, categorisation of symptom domains was performed by age and sex and their distribution.

### Ethical considerations

Data were obtained under umbrella ethics approval for emergency intervention and research in homeless shelters during the South African National State of Disaster, granted by the University of Pretoria’s Faculty of Health Sciences’ Research and Ethics Committee (Ethics Reference No.: 310/2020). The ethical approval for the analysis of the data for the purpose of this study was also obtained from the University of Pretoria’s Faculty of Health Sciences’ Research and Ethics Committee (Ethics Reference No.: 517/2021).

## Results

**FIGURE 1:**
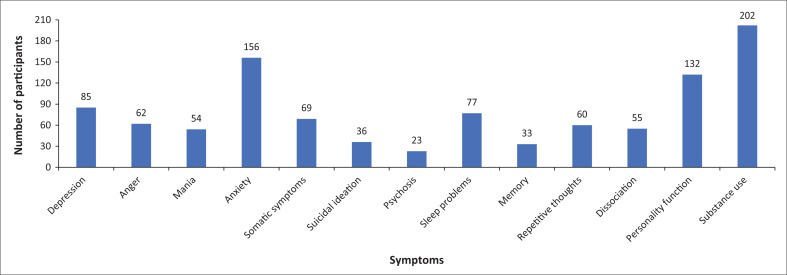
Symptom domain score data.

Figure 1 shows results for 295 participants included in this study. Presence of moderate-to-severe symptoms (indicating that individuals were experiencing symptoms for more than half the days during the past two weeks) was reported as follows: depression (*n* = 85, 29%), anger (*n* = 62, 21%), mania (*n* = 54, 18%), anxiety (*n* = 156, 53%), somatic symptoms (*n* = 69, 23%), suicidal ideation (*n* = 36, 12%), psychosis (*n* = 23, 8%), sleep problems (*n* = 77, 26%), memory (*n* = 33, 11%), repetitive thoughts and behaviours (*n* = 60, 20%), dissociation (*n* = 55, 19%), personality functioning (*n* = 132, 44%) and substance use (*n* = 202, 68%).

The gender distribution among the 295 participants in this study is depicted in Table 3. A total of 265 (89.8%) participants were male, 26 (8.8%) were female and 4 (1.4%) participants identified themselves as ‘other’.

**TABLE 3:** Gender and age distribution.

Variable	Male	Female	Other	Total
**Gender distribution**				
*n*	265	26	4	295
%	89.8	8.8	1.4	-
**Age distribution**				
Mean age	35.0	37.2	36.5	35.2
Standard deviation	8.3	8.1	8.3	8.3
Minimum age	20	22	35	20
Maximum age	75	61	38	75

Table 3 also shows that the age range for the total number of participants is 20–75 years. The mean age for the study population was calculated as 35 years of age with standard deviation (s.d.) of 8.3. The mean age per gender was also calculated to establish whether there was a difference between age distribution among different genders. The mean age for males was 35, for females 37 and for individuals classified as ‘other’ 36 for the s.d. of 8.3.

**FIGURE 2:**
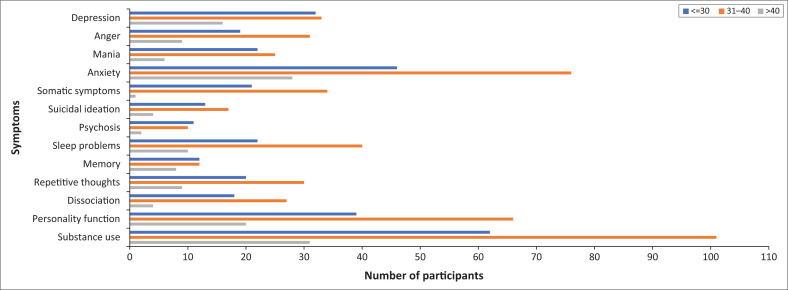
Moderate-to-severe symptom domain prevalence per age group.

Figure 2 demonstrates the prevalence of mental health symptoms as per age category. The age categories depicted were suggested by the biostatistician, according to the age population distribution depicted in Table 3. It is notable that symptom prevalence for the 13 domains was more or less equal across the three age categories, with a few exceptions, these being that the age group 31–40 years has a significantly higher burden relating to anxiety symptoms, sleep problems, as well as substance use symptoms.

## Discussion

In this study, the most prevalent mental health domain symptoms reported as moderate to severe by participants were substance use, anxiety, personality functioning, depression and sleep problems. This emphasises the major burden of mental illness, especially substance use among the homeless community residing in temporary shelters in the CoT during the COVID-19 lockdown.

Stein et al.^19^ found that 30.3% of the general South African population has a lifetime prevalence of a psychiatric disorder. Anxiety disorders (15.8%), followed by substance use disorders (13.3%) and mood disorders (9.8%), were the most prevalent class of disorders. The findings of Stein et al. among the general South African population do not differ significantly from the findings in this study in terms of the symptom domains that are most prevalent. The significantly higher prevalence of anxiety symptoms, sleep problems and substance use symptoms in the age category of 31–40 years perhaps indicates a target age population for intervention strategies.

One of the major non-COVID-19 conditions dealt with in the shelters during the hard lockdown were mental health conditions. Non-governmental organisations such as Médecins Sans Frontières (MSF [Doctors without Borders]) were also involved in supporting the vulnerable homeless population, especially migrants and asylum seekers, with primary health services, mental health services and social support services.^20^ A psychologist working for MSF reported a decrease in visits for mental health services to one of their facilities in Tshwane during the lockdown period. She observed in practice that the number of patients missing psychotherapy appointments was higher during the initial stages of lockdown because of various reasons, including the lockdown itself, fear of contracting the virus, unemployment and transport difficulties.^20.21^

Opioid-dependent homeless people formed a large group of the almost 2000 residents initially housed at the Caledonian stadium in the inner city of Tshwane by the CoT during the early period of the level 5 COVID-19 lockdown.^11^ Withdrawal management through opioid substitution therapy was provided by COSUP in conjunction with stakeholders. Despite the emergency response for the homeless community initially being overwhelming to the team at hand, harm reduction services are increasingly proving to be successful.^11^ Scheibe et al. identified the significant improvement in quality of life with an increase in the mental composite score in individuals retained in an opioid substitution maintenance programme.^22^

Initially gender was only distinguished as male or female, but the importance was recognised to offer participants the option to identify their gender (rather than biological sex) as ‘other’ – thereby including individuals identifying as transgender and gender non-conforming individuals. Although only 1.4% of this population studied presented as ‘other’, Downing and Przedworski found in their study^23^ that all transgender groups, especially gender non-conforming populations, have a high burden of disabilities, higher odds of chronic illness and poor mental health, reminding us to consider this group when providing health and social services within the homeless community.

This study, supported by findings of Scheibe et al.,^7^ demonstrates that males more commonly engage in using harmful substances than females.^19^ The nature of the gender distribution in shelters, by virtue of capacitance, accommodated more males than females. Even though some larger shelters were set up to accommodate both males and females, the temporary shelter capacity still reflected a larger male population. It is possible to explain this: homeless women who use substances face a range of barriers and are often less visible. These barriers include the fear of child custody issues with social service involvement and contact with the criminal justice system, stigmatisation and judgement by society and healthcare providers and limited social support (including financial and family support).^24,25^

Service-provision to the street-homeless community, such as the treatment of chronic diseases and mental health conditions (including substance use disorder and acute withdrawal), has been affected by multiple reasons. These include a lack of education, stigmatisation and the fact that proof of identity, which is often not available, is a requirement at most health institutions.^4^

Identifying substance use as a major burden of mental illness in the homeless population of the CoT indicates areas for focused service provision. Ways to achieve specialised service provision for this population are discussed by Heese et al. in Chapter 6 of ‘Facing Homelessness.’^26^ From a COPC perspective, embedding peers and community health workers in the care team for the homeless plays an important role. Furthermore, local organisations such as NGOs play a cardinal role in the continuum of care for the homeless. It is important that role players who provide services within the community are trained in mental health services and support as well as substance use and harm reduction practices. Appropriate support and referral routes should also be in place where the need for inpatient based medical intervention is necessary. Practical guidelines for communities and local organisations to support the homeless, specifically with attention to healthcare, include understanding the context, reaching out and building relationships (knowing people by name and developing communities of care), building a continuum of care and housing interventions.^26^

Studies looking at the uptake and retention of individuals in maintenance programmes such as COSUP after the closure of shelters will be helpful in showing treatment successes or failures. Further research is required to better understand and overcome the barriers street-homeless people have in accessing health and social services.

This study could contribute to future research by serving as a preliminary study of quantitative nature. Important questions arise from these results with regard to why and how certain findings are as they were found to be. This could be answered by future qualitative studies.

Is substance use the reason for the presence of other mental health symptoms or are mental health symptoms because of other circumstances the cause for individuals turning to substances? While these are important questions, this study was not designed to answer these questions and perhaps gives rise to opportunities for future research to be conducted.

### Strengths and limitations

Strengths of this study include the identification of important mental health needs in this vulnerable community. In-person service provision on a daily basis where homeless people were gathered created the ideal circumstances for assessment and interventions.

The DSM-5 CC Symptoms Measure as a data collection tool is understood to be used as a tracking tool for symptom severity and treatment progress, not necessarily as a screening tool. At the time of the study, the intention was to identify prevalent symptom domains and offer follow-up mental health services to participants. This was unfortunately not possible because of insufficient staff for mental health service provision, high burden of services required and high turnover of residents moving in and out of shelters with poor continuum of care as a result of situational circumstances.

Other limitations of this study include the convenience sampling method used. This method was chosen because of geographic distribution and the logistical implications to reach all shelters, high turnover of residents moving in and out of shelters, as the lockdown levels moved down, temporary nature of the shelter setup with shelters closing down and constraints with regard to resources and service provision to a previously difficult accessible population.

This study was conducted where people facing homelessness were accommodated in shelters, provided with food, basic health services and opioid substitution therapy. The possibility exists that results would not be the same outside of the shelter setup.

Findings of this study might be affected by the fact that severely ill mental health users who did not have capacity to consent to this study could not participate although a few were identified and referred for urgent hospital care. Whether these patients were previously known mental healthcare users was not further investigated.

## Conclusion

The South African national lockdown regulations mandated the state to provide shelter to the vulnerable street-based homeless population during that time. The major mental health concern among street-homeless people in CoT, as shown by this study, is substance use. The COVID-19 lockdown created the opportunity for primary care services, including mental health services and OST, to be provided to an otherwise under-served community. Community-oriented and person-centred health services with clear care-coordination pathways seem to be more effective in service provision to the street-homeless community and bring services to the population at risk.
